# Detection of Iris Presentation Attacks Using Feature Fusion of Thepade’s Sorted Block Truncation Coding with Gray-Level Co-Occurrence Matrix Features

**DOI:** 10.3390/s21217408

**Published:** 2021-11-08

**Authors:** Smita Khade, Shilpa Gite, Sudeep D. Thepade, Biswajeet Pradhan, Abdullah Alamri

**Affiliations:** 1 Symbiosis Institute of Technology, Symbiosis International, Deemed University, Pune 412115, India; smita.khade.phd2020@sitpune.edu.in; 2 Symbiosis Centre for Applied Artificial Intelligence, Symbiosis International, Deemed University, Pune 412115, India; 3 Pimpri Chinchwad College of Engineering, Savitribai Phule Pune University (SPPU), Pune 411044, India; sudeepthepade@gmail.com; 4 Centre for Advanced Modelling and Geospatial Information Systems (CAMGIS), Faculty of Engineering and Information Technology, University of Technology, Sydney, NSW 2007, Australia; 5 Earth Observation Centre, Institute of Climate Change, Universiti Kebangsaan Malaysia, Bangi 43600, Malaysia; 6 Department of Geology & Geophysics, College of Science, King Saud University, P.O. Box 2455, Riyadh 11451, Saudi Arabia; amsamri@ksu.edu.sa

**Keywords:** iris images, liveness detection, TSBTC, GLCM, machine learning, feature extraction, feature fusion

## Abstract

Iris biometric detection provides contactless authentication, preventing the spread of COVID-19-like contagious diseases. However, these systems are prone to spoofing attacks attempted with the help of contact lenses, replayed video, and print attacks, making them vulnerable and unsafe. This paper proposes the iris liveness detection (ILD) method to mitigate spoofing attacks, taking global-level features of Thepade’s sorted block truncation coding (TSBTC) and local-level features of the gray-level co-occurrence matrix (GLCM) of the iris image. Thepade’s SBTC extracts global color texture content as features, and GLCM extracts local fine-texture details. The fusion of global and local content presentation may help distinguish between live and non-live iris samples. The fusion of Thepade’s SBTC with GLCM features is considered in experimental validations of the proposed method. The features are used to train nine assorted machine learning classifiers, including naïve Bayes (NB), decision tree (J48), support vector machine (SVM), random forest (RF), multilayer perceptron (MLP), and ensembles (SVM + RF + NB, SVM + RF + RT, RF + SVM + MLP, J48 + RF + MLP) for ILD. Accuracy, precision, recall, and F-measure are used to evaluate the performance of the projected ILD variants. The experimentation was carried out on four standard benchmark datasets, and our proposed model showed improved results with the feature fusion approach. The proposed fusion approach gave 99.68% accuracy using the RF + J48 + MLP ensemble of classifiers, immediately followed by the RF algorithm, which gave 95.57%. The better capability of iris liveness detection will improve human–computer interaction and security in the cyber-physical space by improving person validation.

## 1. Introduction

Automatic access to a system by a genuine person has become very simple in the information era. For automated system access, validation of user identity is crucial. Biometric authentication systems are computer-based systems that use biometric traits to verify a user’s identity. The biometric authentication system has more advantages over other password-based conventional authentication mechanisms [1]. The biometric system eliminates the need to remember a password or pin or keep a card in possession. The conventional security system cannot differentiate between real persons and impostors, those who can unethically access a program [2]. For security-critical cyber applications, biometric authentication may also be considered an additional layer of authentication along with existing conventional authentication modes [3]. As the iris has a complex texture and unique features, it is widely used to identify and authenticate a person in most applications [2], e.g., in the Aadhaar card project to identify India’s citizens. The Amsterdam airport and United States–Canadian border [4] use iris-based authentication. Compared to fingerprints and facial recognition, iris-based authentication enables a more reliable contactless detection of a user. The contactless approach helps to prevent the spread of viruses and diseases such as COVID-19. Even though the iris has a unique textural pattern, an impostor might counterfeit it.

People usually attack the biometric system to gain access to other people’s accounts or to hide their true identity. The iris detection system can be easily spoofed by using different types of spoofing attacks. Table 1 shows the iris presentation attacks used that are found in the literature.

Analyzing threat and vulnerability is crucial for securing the biometric system. The challenging threat of spoofing a biometric authentication system is mitigated with liveness detection of acquired biometric traits before authentication [9].

The critical contributions of the research work presented here are as follows:

- Development of Thepade’s sorted block truncation coding (TSBTC) and gray-level co-occurrence matrix (GLCM) iris image data as features for the first time in iris liveness detection (ILD).
- Implementation of the fusion of best TSBTC N-ary global features with GLCM local features from an iris image, for the first time in ILD.
- Performance analysis of ML classifiers and ensembles to finalize the best classifier for ILD.
- Validating the performance of the proposed ILD method across various existing benchmark datasets and techniques.

The paper is organized as follows: Section 2 briefly reviews the related literature, Section 3 presents an overview of existing methods, and Section 4 presents the proposed ILD method. The experiment details, observed results, and inferences drawn from the results are discussed in Section 5. The concluding remarks and future research directions are discussed in Section 6.

## 2. Related Work

Many attempts have been made to detect the liveness of the sensed biometric traits before they are authenticated. A few prominent approaches are discussed in this section. Kaur et al. [10] used a rotation-invariant feature set consisting of Zernike moments and polar harmonic transforms that extract local intensity variations to detect iris spoofing attacks. The spoofing attacks on various sensors also have a considerable effect on the overall efficiency of a system. A system can detect only print and contact lens attacks. They used Hough transform and GLCM to extract features from iris images. These extracted features were passed for discriminant analysis (DA), used as a classification tool for differentiating live images from spoofed ones.

Agarwal et al. [11] used fingerprints and iris identity for liveness detection. The standard Haralick’s statistical features based on the GLCM and neighborhood gray-tone difference matrix (NGTDM) generate a feature vector from the fingerprint. Texture features from the iris are used to boost the performance of a system. They used a standard dataset to test if the performance of this model is better than the existing model. In the existing system, GLCM has a huge feature vector size.

In a recent paper, Jusman et al. (2020) [12] compared the proposed approach with other existing approaches and proved that the proposed approach performs better, with 100% accuracy. The limitation of this study is that the authors followed a traditional approach of segmentation, normalization, and feature extraction, which is a complex and time-consuming task. Subsequently, Khuzani et al. [13] extracted the shape, density, FFT, GLCM, GLDM, and wavelet features from iris images. In total, 2000 iris images from the CASIA-Iris-Interval dataset are used for implementation. The highest accuracy of 99.64% was achieved by using a multilayer neural network.

Agarwal et al. (2020) used a feature descriptor, i.e., local binary hexagonal extreme pattern, for fake iris detection [14]. The proposed descriptor exploits the relationship between the center pixel and its hexa neighbor. The hexagonal shape using the six-neighbor approach is preferable to the rectangular structure due to its higher symmetry. This approach’s limitation is that it covers only print and contact lens attacks and is highly complex [14]. Thavalengal et al. (2016) [15] developed a smartphone system that captures RGB and NIR images of the eye and the iris. Pupil localization techniques with distance metrics are used for detection. For feature vector generation, 4096 elements are considered, which are extensive. Even though the authors claimed a reasonable liveness detection rate, they worked with a real-time database.

TSBTC has been used many times in the literature in other domains, but none of the studies has identified iris liveness detection using TSBTC. Some of the studies from other domains are discussed here. Dewan et al. (2021) used TSBTC to retrieve images from datasets using the key points extraction method [16]. Chaudhari et al. (2021) used a fusion of TSBTC and Sauvola thresholding features [17]. With the help of multiple classifiers, including SVM, Kstar, J48, RF, RT, and ensembles, the authors achieved good accuracy. To enhance image classification, Thepade et al. (2018) used TSBTC with feature-level fusion of Niblack thresholding and SVM, RF, Bayes net, and ensembles of classifiers [18].

In their work, Fathy and Ali (2018) did not consider the segmentation and normalization phases typically used in fake iris detection systems [8]. Wavelet packets (WPs) are used to break down the original image into a wavelet. They claimed 100% accuracy, but it does not work with all types of attacks, and it covered only limited spoofing attacks. Hu et al. (2016) performed ILD using regional features [19]. Regional features are designed based on the relationship of the features with the neighboring regions. During an experiment, Hu used 144 relational measures based on regional features. Czajka (2015) designed a liveness detection system using pupil dynamics [20]. In this system, pupil reaction is measured with the help of sudden changes in light intensity. If the eye reacts to light intensity changes, then the eye is live; otherwise, it is fake. Linear and non-linear support vector machines are used to classify natural reactions and spontaneous oscillations in this work. The limitation of the system measures diverse functions, which take time. The data used in this analysis do not include any measurements from older people, so there is inaccuracy in the observation [20].

Naqvi et al. (2020) developed a system to detect accurate ocular regions, such as the iris and sclera [21]. This system is based on the convolutional neural network (CNN) model with a lite-residual encoder–decoder network. Average segmentation error is used to evaluate the segmentation results. Publicly available databases are considered for evaluating the system. Kimura et al. (2020) designed a liveness detection system using CNN, which improves the model’s accuracy by tuning hyperparameters [22]. To measure the performance of the system, attack presentation classification error rate (APCER) and bonafide presentation classification error rate (BPCER) are used. The hyperparameters considered in this paper are the number of epochs (max), batch size, learning rate, and weight decay hyperparameters. This system works only for print and contact lens attacks.

Lin and Su (2019) developed a face anti-spoofing and liveness detection system using CNN [23]. The image is resized to 256 * 256, and RGB and HSV color spaces are used. The author claims better iris liveness prediction [23]. Long and Zeng (2019) identified ILD with the help of the BNCNN architecture with 18 layers. The batch normalization technique is used in BNCNN to avoid the problem of overfitting and gradient disappearing during training [24].

Dronky et al. (2019) [25] observed from the literature that many researchers do not identify all iris attacks. So, from the existing literature, it is observed that researchers have worked on a few iris attacks, and a prominent feature vector size is considered. Table 2 summarizes the literature review in ascending order of the year of publication.

## 3. Proposed Iris Liveness Detection Using a Feature-Level Fusion of TSBTC and GLCM

**Figure 1:**
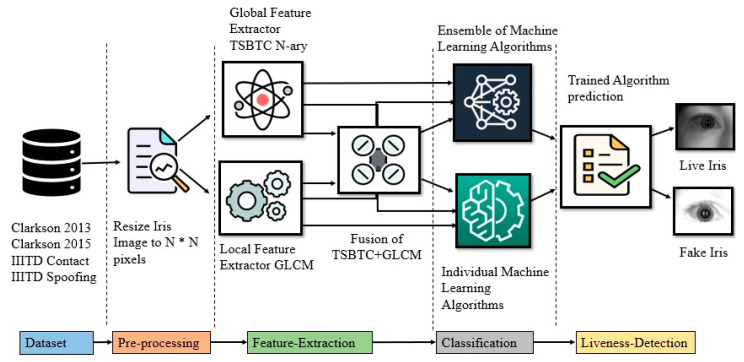
Block diagram of the proposed ILD using feature fusion of TSBTC with GLCM features.

Iris recognition system is susceptible to many security challenges. These vulnerabilities make a system less reliable for highly secured applications [3]. This paper attempts ILD using the feature-level fusion of GLCM and TSBTC features of iris images, which detect whether the iris is live or fake. The proposed approach avoids any preprocessing, such as segmentation, normalization, and localization, conventionally used by the methods proposed in the literature, making the proposed approach swifter and relatively easier [15]. The only preprocessing done in the proposed approach is resizing the iris image to square size. Figure 1 shows the block diagram of the ILD system. The proposed system is divided into four phases: iris image resizing, feature formation, classification, and ILD.

### 3.1. Resizing

Iris preprocessing plays a vital role in ILD. In the proposed algorithm, two iris preprocessing approaches are followed. Images are acquired using four different standard datasets, so each dataset uses a different size of images to be stored. During preprocessing, the original images are normalized to the size 128 * 128, which maintains integrity throughout the experiment. While images capture different datasets using different sensors, some sensors (e.g., LG, Congent, Vista) capture images in the RGB format, and some (e.g., LG, Dalsa) capture them in the grayscale format. To maintain uniqueness, images are converted into the grayscale format.

### 3.2. Feature Formation and Fusion

In the proposed method, feature fusion is attempted with the help of GLCM and Thepade’s SBTC applied on iris images.

#### 3.2.1. GLCM

**Figure 2:**
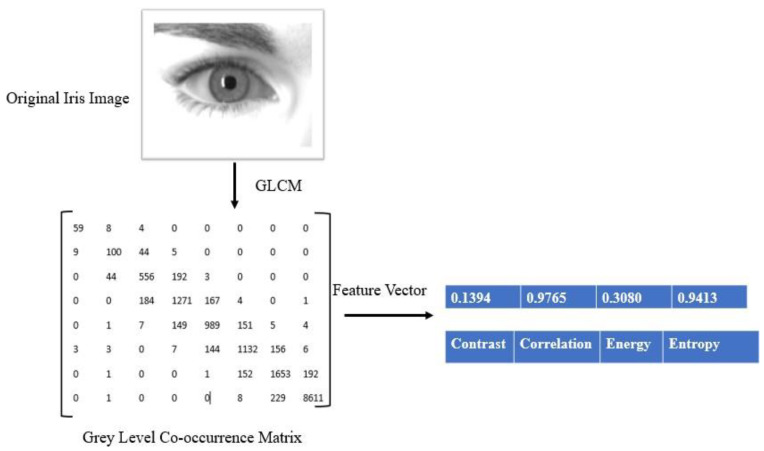
GLCM feature extraction from an iris image.

The statistical distribution information of the gray-level value of an image is generated by GLCM [27,28]. GLCM is applied to a resized iris image. Figure 2 shows feature formation using GLCM. We computed four features: contrast, energy, entropy, and correlation using GLCM. These are given by:

*Energy*: Local gray-level consistency represents energy as expressed in Equation (1), which is high in similar pixels.

(1) $$\;Energy=\underset{i,j}{\sum }P\;i,j{\;}^{2}$$

*Entropy*: The following image entropy equation is used to describe an image’s randomness. The greater the entropy, the more difficult it is to arrive at any conclusion from the data.

(2) $$Entropy=-\underset{i,j}{\sum }P\;i,j\;log2\;P\;i,j\;$$

*Contrast*: As expressed in Equation (3), contrast is used to evaluate the intensity difference between the reference pixel and its neighbor. The low-intensity value represents the GLCM’s poor contrast.

(3) $$Contrast=\underset{i,j}{\sum }{\left|i-j\right|}^{2}\;Pi,j$$

*Correlation*: Equation (4) represents the linear dependency of gray-level values in the co-occurrence matrix.

(4) $$Correlation=\underset{i,j}{\sum }\frac{\left(i-u_{i}\right)\left(j-u_{j}\right)Pi,j\;}{\sigma i\sigma j}$$

These four features for one image are taken into consideration. The 10 cross-validation technique is used for the correct estimation of accuracy.

#### 3.2.2. TSBTC

**Figure 3:**
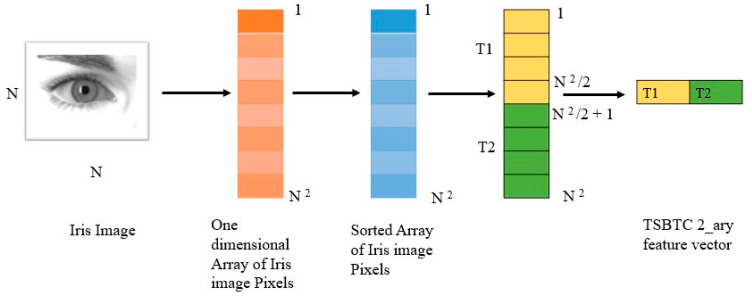
TSBTC feature extraction from an iris image.

Let the iris image be I (*r*,*c*) of size *r* × *c* pixels, grayscale. The TSBTC [29,30] feature vector of N-ary may be considered as [T1, T2, Tn]. Here, Ti indicates the *i*^th^ cluster centroids of the grayscale image using TSBTC N-ary. In TSBTC 2-ary, for iris image I (*r*,*c*) of size *r* × *c* pixels, the grayscale image is converted into a one-dimensional array sorted as sortrows. Using this one-dimensional sorted array, the TSBTC-2ary feature vector is computed as [T1, T2], as shown in Equations (5) and (6). Figure 3 shows how features are extracted using TSBTC.

(5) $$T1=\frac{2}{r\times c\;}\overset{r\times c}{\underset{p=1}{\sum }}Sort\left(p\right)$$

(6) $$T2=\frac{2}{r\times c\;}\overset{r\times c}{\underset{p=1+\frac{r\times c}{2}\;}{\sum }}Sort\left(p\right)$$

Here is the proposed ILD. TSBTC has experimented with all 10 variations of TSBTC 2-ary, 3-ary, 4-ary, 5-ary, 6-ary, 7-ary, 8-ary, 9-ary, 10-ary, and 11-ary with a resized iris image. These extracted features are passed to classifiers and ensembles of classifiers to train them.

#### 3.2.3. Fusion of TSBTC and GLCM

**Figure 4:**
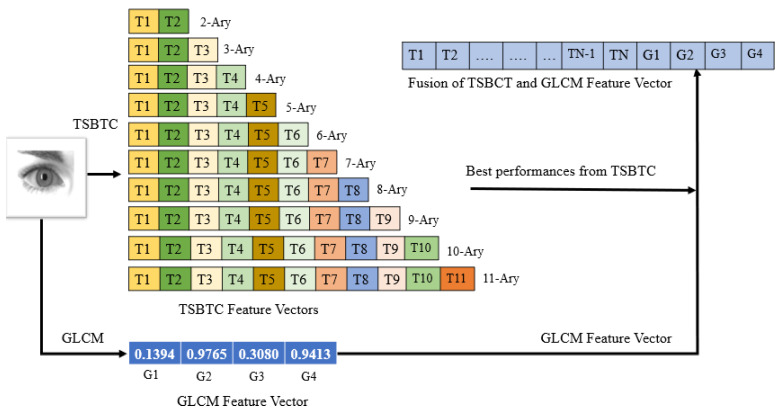
Fusion of GLCM and TSBTC feature vectors from iris image.

The best performance from TSBTC N-ary and local-level GLCM features are concatenated to get the feature-level fusion for ILD. Here, both fusions are considered TSBTC 10-ary + GLCM and TSBTC 11-ary + GLCM. Let the grayscale iris image be I (*r*,*c*) of size *r* × *c* pixels. The fusion of the feature vector of TSBTC N-ary and GLCM can be represented as [T1, T2, Tn, G1, G2, G3, G4]. Figure 4 displays how feature vectors are formed by taking the fusion of GLCM and TSBTC. Individual feature vector elements can be extracted using the mathematical model elaborated in Section 3.2.1 and Section 3.2.2

### 3.3. Classification and Iris Liveness Detection

The proposed approach of ILD uses different ML classifiers with an ensemble combination. The tenfold cross-validation approach is used for training these classifiers for ILD. Tenfold cross-validation is one of the best approaches for training ML classifiers. It provides all samples from the dataset a chance to be used as training or test data, resulting in a trained classifier that is less biased. The ML classifiers employed here are support vector machine (SVM), naïve Bayes (NB), random forest (RF), random tree, and J48, with ensembles of a few of the ML classifiers. Majority of voting logic is used here for creating the ensembles of ML classifiers.

*SVM*—Its main aim is that in an N (number of features)-dimensional space, it must find a hyperplane that distinctly classifies the data points. The objective of an SVM is to find a plane with the maximum distance between data points of classes [31].

*J48*—It is a classification algorithm. It is the decision tree-based classification algorithm as that of the decision tree classifier [31].

*Random Forest*—It takes the mean prediction of the individual trees formed from an ensemble of various decision trees. This algorithm makes sure that the decision tree classifier’s drawback of overfitting the training data is overcome [31].

*Random Tree*—Random tree is a parameter-based supervised learning algorithm with continuous data splitting. The random tree algorithm is similar to the decision tree algorithm, which is made by selecting random features [32].

*Naïve Bayes*—This algorithm is based on the theorem of Bayes and is a collection of classification algorithms. It is a family of algorithms, as all of them share a common objective. It predicts probabilities belonging to a class for each data point [32]

*Ensemble method*—It is always better to use multiple models simultaneously on a single set for classification rather than just a single model. This method is called ensemble learning [17]. A model is trained by using different classifiers, and the final output is an ensemble of those classifiers. Majority voting logic has been used for an ensemble of ML classifiers in the proposed method.

## 4. Experimental Set-Up

The experiments were performed using an Intel (R) Core (TM) i3-6006U CPU @ 2.0 GHz, 12 GB RAM, and 64-bit operating system with MATLAB R2015a as a programming platform. The experimentation code is available on request. The datasets used for experimental explorations of the proposed approach of ILD are Clarkson LiveDet2013, Clarkson LiveDet2015, IIITD Contact Lens, and IIITD Combined Spoofing.

### 4.1. Description of the Dataset

The detailed description of the four standard and publicly available datasets is as follows.

**Figure 5:**
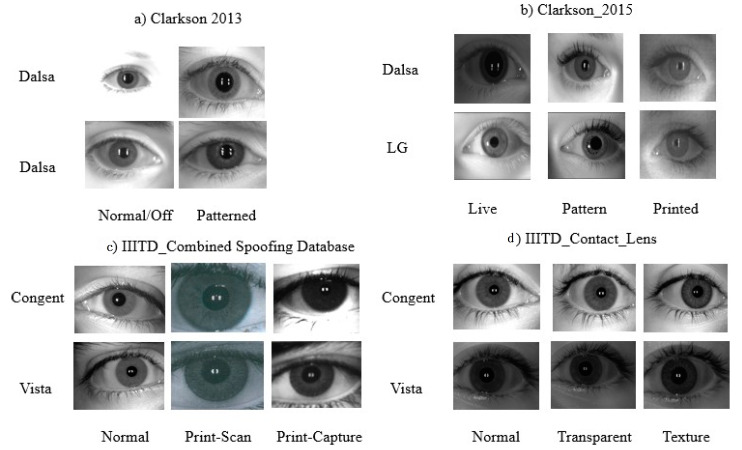
Sample iris images from an iris dataset.

- Clarkson LivDet2013—Clarkson LivDet2013 dataset has around 1536 iris images [33]. This dataset is separated into training and testing sets. To acquire images, the Dalsa sensor is used. During this experiment, the training set images are used. Table 3 shows details related to the dataset, the sensors used to acquire images, and the number of images used during this experiment. Figure 5 shows samples of images from the dataset.
- Clarkson LivDet2015—Images used in this dataset are captured using Dalsa and LG sensors [34]. Images are divided into three categories: live, pattern, and printed. In total, 25 subjects are used for live images and patterns are printed; 15 subjects each are used. The whole dataset is partitioned into training and testing.
- IIITD Combined Spoofing Database—Images used in this dataset are captured using two iris sensors, Cogent and Vista [35]. The images are divided into three categories: normal, print-scan attack, and print-capture attack.
- IIITD Contact Lens—Images used in this dataset are captured using two iris sensors, Cogent dual iris sensor and Vista FA2E single iris sensor [36,37]. The images are di-vided into three categories: normal, transparent, and colored. In total, 101 subjects are used. Both left and right iris images of each subject are captured; therefore, there are 202 iris classes.

### 4.2. Performance Measures

To compare the performance of all the investigated variations of the proposed ILD method, accuracy, recall, F-measure, and precision are used as performance metrics.

**Figure 6:**
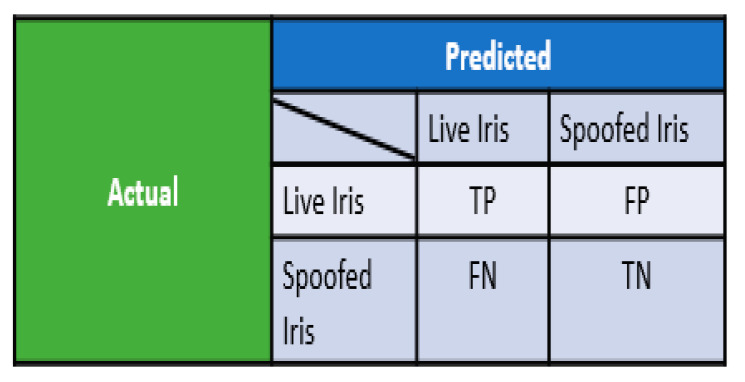
Confusion matrix for iris liveness detection.

Let TP, TN, FP, and FN, respectively, be the true positive, true negative, false positive, and false negative of the ILD. TP indicates the data samples that are predicted as live iris and are actually live samples. TN gives the data samples detected as spoofed iris and actually spoofed iris samples. FP indicates the samples identified as live but are fake. FN shows the data samples detected as spoofed but are live iris samples. The confusion matrix is shown in Figure 6. Equations (7)–(13) were used to calculate the accuracy, precision, recall, F-measure, attack presentation classification error rate (APCER) [38], normal presentation classification error rate (NPCER)*,* and average classification error rate (ACER)*,* respectively.

(7) $$\mathrm{Accuracy}=\frac{\mathrm{TP}\;+\;\mathrm{TN}}{\mathrm{FP}\;+\;\mathrm{FN}\;+\;\mathrm{TP}\;+\;\mathrm{TN}}$$

(8) $$\mathrm{Precision}=\frac{\mathrm{TP}}{\mathrm{FP}\;+\;\mathrm{TP}}$$

(9) $$\mathrm{Recall}=\frac{\mathrm{TP}}{\mathrm{TP}\;+\;\mathrm{TN}}$$

(10) $$F-\mathrm{measures}=2*\;\frac{\mathrm{Precision}*\;\mathrm{Recall}}{\mathrm{Precision}\;+\;\mathrm{Recall}}$$

(11) $$\;\mathrm{APCER}\;=\frac{\mathrm{FP}}{\mathrm{FP}\;+\;\mathrm{FN}}$$

(12) $$\mathrm{NPCER}\;=\frac{\mathrm{FN}}{\mathrm{FN}\;+\;\mathrm{TP}}$$

(13) $$\;\mathrm{ACER}\;=\frac{\mathrm{APCER}\;+\;\mathrm{NPCER}}{2}$$

## 5. Results

This section is organized into three subsections. Section 5.1 presents the results and graphs of the TSBTC approach. Section 5.2 presents the results of the GLCM technique. The fusion of TSBTC and GLCM is discussed in Section 5.3.

### 5.1. TSBTC Results

The proposed approach of ILD experiments with four benchmark datasets. Accuracy, recall, precision, and F-measure are used as performance metrics to evaluate the variants of the proposed approach of ILD. With 128 ×128 iris images, TSBTC experiments with all 10 varieties of the TSBTC 2-ary, 3-ary, 4-ary, 5-ary, 6-ary, 7-ary, 8-ary, 9-ary, 10-ary, and 11-ary. These extracted features are passed to classifiers and ensembles of classifiers to train them.

**Figure 7:**
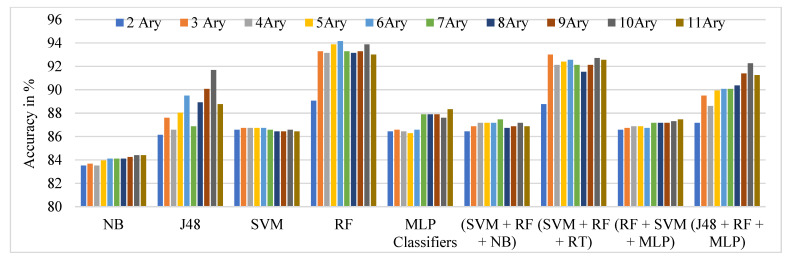
Performance evaluation of TSBTC N-ary global feature variations for the specific ML classifiers in the proposed approach of ILD for Clarkson 2013 dataset using percentage accuracy.

The performance comparison of the TSBTC N-ary global features considered for specific ML classifiers in the proposed approach of ILD tested on the Clarkson 2013 dataset is shown in Figure 7. It can be observed that 10-ary TSBTC outperforms other N-ary TSBTC approaches for all classifiers for the Clarkson 2013 dataset.

From Table 4, it is observed that the highest ILD accuracy comes to around 94.16% with 6-ary TSBTC using the RF classifier, immediately followed by an ensemble of RF + SVM + RT classifiers. The underlined values indicate the highest obtained recognition rates.

**Figure 8:**
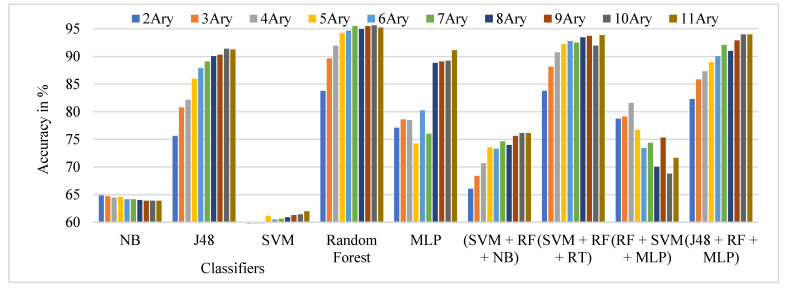
Performance evaluation of TSBTC N-ary global feature variations for the specific ML classifiers in the proposed approach of ILD for the Clarkson 2015 dataset using percentage accuracy.

The performance comparison of the TSBTC N-ary global features considered for specific ML classifiers in the proposed approach of the ILD tested on the Clarkson 2015 dataset is shown in Figure 8. As per comparison, 11-ary TSBTC outperforms other N-ary TSBTC approaches for the Clarkson 2015 dataset for all classifiers.

From Table 5, it is observed that the highest observed ILD accuracy comes to around 95.64% with 10-ary TSBTC using the RF classifier, immediately followed by an ensemble of RF + SVM + RT classifiers.

**Figure 9:**
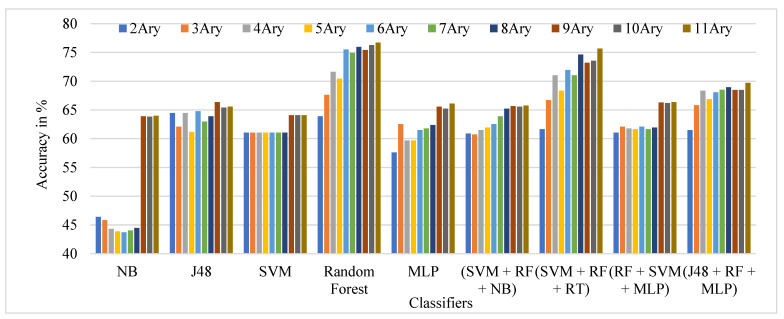
Performance evaluation of TSBTC N-ary global feature variations for the specific ML classifiers in the proposed approach of ILD for the IIITD Contact dataset using percentage accuracy.

The performance comparison of the TSBTC N-ary global features considered for specific ML classifiers in the proposed approach of ILD tested on the IIITD Contact dataset is shown in Figure 9. It can be observed that 11-ary TSBTC outperforms other N-ary TSBTC approaches for all classifiers for the IIITD Contact dataset.

From Table 6, it is observed that the highest observed ILD accuracy comes to around 76.73% with 11-ary TSBTC using the random forest classifier, immediately followed by an ensemble of RF + SVM + RT classifiers.

**Figure 10:**
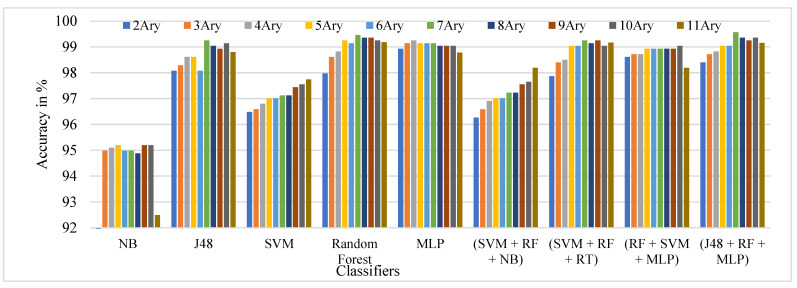
Performance evaluation of TSBTC N-ary global feature variations for the specific ML classifiers in the proposed approach of ILD for the IIITD Combined Spoofing dataset using percentage accuracy.

Figure 10 shows the performance comparison of the global features of TSBTC N-ary considered for specific ML classifiers in the proposed approach of ILD tested on the IIITD Combined Spoofing dataset. It can be observed that 10-ary TSBTC outperforms other N-ary TSBTC approaches for all classifiers for the IIITD Combined Spoofing dataset.

From Table 7, it is observed that the highest observed ILD accuracy comes to around 99.57% with 7-ary TSBTC using an ensemble of J48 + RF + MLP classifiers, immediately followed by RF classifiers.

### 5.2. GLCM Results

In the proposed ILD, features are extracted using GLCM by using equations explained in Section 3.2.1. These extracted features are passed to classifiers and ensembles of classifiers to train them.

**Figure 11:**
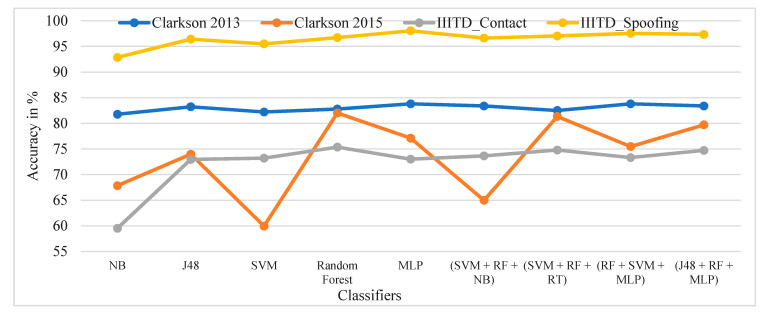
Performance evaluation of GLCM local features for the specific ML classifiers in the proposed approach of ILD across all datasets using percentage accuracy.

Figure 11 shows the performance comparison of the GLCM local features considered for specific ML classifiers in the proposed approach of ILD tested across all datasets. Here, it can be observed that random forest and ensembles of RF + SVM + MLP give the best performance across all datasets.

**Figure 12:**
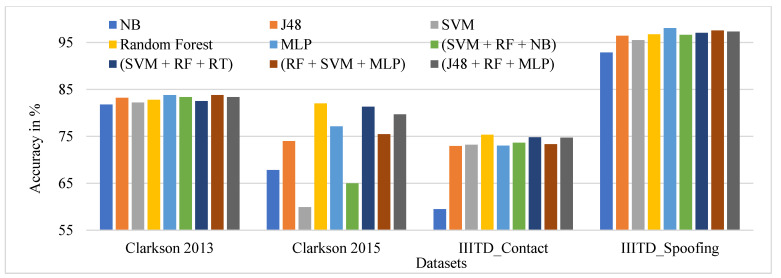
Performance evaluation of GLCM local features across all datasets for the specific ML classifiers in the proposed approach of ILD using percentage accuracy.

The performance evaluation of GLCM local features across all datasets for specific ML classifiers in the proposed approach of ILD using percentage accuracy is shown in Figure 12. The graph shows that IIITD Combined Spoofing gives good performance across all classifiers and ensembles of classifiers.

### 5.3. Fusion of TSBTC and GLCM Results

The best performance from TSBTC N-ary and GLCM local-level features are concatenated to get the feature-level fusion for ILD. Here, both fusions are considered, TSBTC 10-ary + GLCM and TSBTC 11-ary + GLCM.

**Figure 13:**
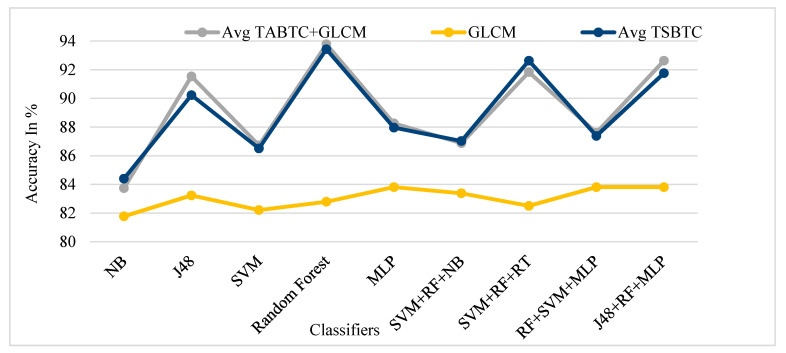
Performance evaluation of TSBTC, GLCM, and fusion of TSBTC and GLCM local features for the specific ML classifiers in the proposed approach of ILD for the Clarkson 2013 dataset using percentage accuracy.

**Figure 14:**
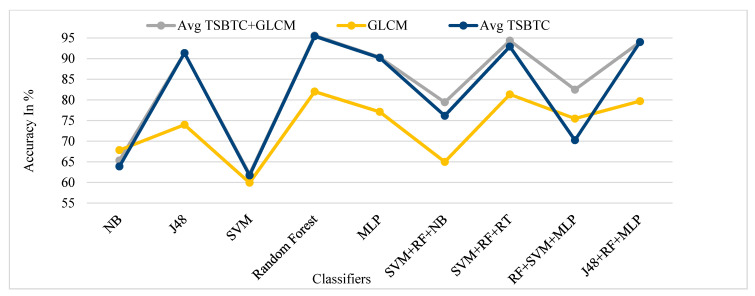
Performance evaluation of TSBTC, GLCM, and fusion of TSBTC and GLCM local features for the specific ML classifiers in the proposed approach of ILD for the Clarkson 2015 dataset using percentage accuracy.

**Figure 15:**
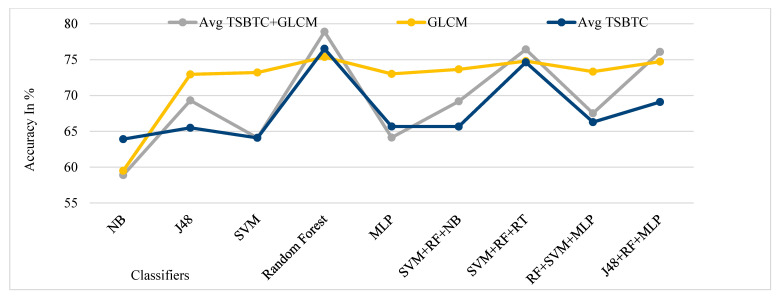
Performance evaluation of TSBTC, GLCM, and fusion of TSBTC and GLCM local features for the specific ML classifiers in the proposed approach of ILD for IIITD Contact.

**Figure 16:**
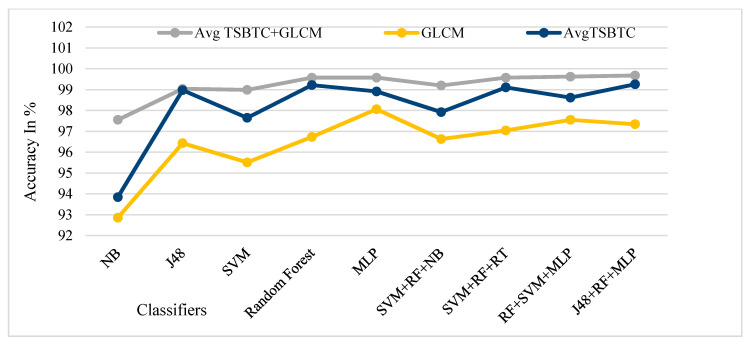
Performance evaluation of TSBTC, GLCM, and fusion of TSBTC and GLCM local features for the specific ML classifiers in the proposed approach of ILD for IIITD Combined Spoofing dataset.

Figure 13, Figure 14, Figure 15 and Figure 16 show the performance comparison of TSBTC, GLCM, and the fusion of the global features of TSBTC N-ary and local features of GLCM for specific ML classifiers in the proposed approach of ILD tested on Clarkson 2013, Clarkson 2015, IIITD Contact, and IIITD Combined Spoofing dataset, respectively. Here, an observed fusion of TSBTC and GLCM gives the best performance across all datasets.

From Table 8, it is observed that the highest ILD accuracy comes to around 93.78% with the fusion of TSBTC’s highest performance with GLCM features using the RF classifier for the Clarkson 2013 dataset. The highest accuracy comes to around 95.57%, with the fusion of TSBTC’s highest performance with GLCM features using the RF classifier for the Clarkson 2015 dataset. For the IIITD Contact dataset, the highest accuracy comes to around 78.88%, with the fusion of TSBTC’s highest performance with GLCM features by using the RF classifier. The highest observed ILD accuracy comes to around 99.68%, with the fusion of TSBTC’s highest performance with GLCM features using RF and an ensemble of J48 + RF + MLP classifiers for the IIITD Combined Spoofing dataset.

**Figure 17:**
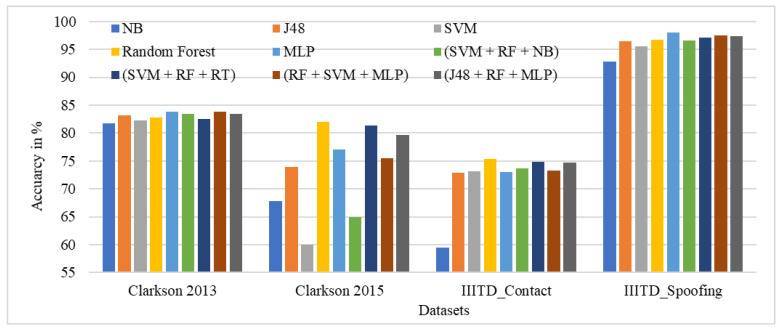
Performance evaluation of the fusion of TSBTC and GLCM local features across all datasets for the specific ML classifiers in the proposed approach of ILD.

Figure 17 shows the performance comparison of the fusion of global features of TSBTC N-ary and GLCM local features considered for specific ML classifiers in the proposed approach of ILD tested on all datasets. Here, the fusion of TSBTC and GLCM gives the best performance for all datasets used during the experiments. The highest accuracy achieved is 99.68% for the IIITD Combined Spoofing dataset, which shows that it outperforms others.

It is observed from Table 9 that ensembles of J48 + RF + MLP classifiers give the highest accuracy (99.68%) and lowest rate of ACER (0.48%) using the fusion of TSBTC and GLCM local features in the proposed approach of ILD used with the IIITD Combined Spoofing dataset.

## 6. Discussions

Based on the current experiments, GLCM and TSBTC are the widely utilized image feature extraction methods. Thepade’s sorted block truncation coding (TSBTC) has been employed in various image classification applications. For the very first time, TSBTC has been designed to assess iris presentation attacks. The feature vector generated by the methods described in Section 3.2 is then supplied as an input to machine learning and ensembles of classifiers described in Section 3.3 using the Weka tool. Groupings of TSBTC and GLCM features are used to achieve feature-level fusion. For testing purposes, four standard datasets are used: Clarkson 2013, Clarkson 2015, IIITD Contact, and IIITD Combined Spoofing; additionally, databases such as Clarkson 2017 and CASIA can be examined in the future.

GLCM, a local feature extraction approach that has delivered an excellent average classification accuracy, as stated in Section 5.2, has been seen in investigations of iris presentation attack detection. As explained in Section 5.1, TSBTC has shown better accuracy versus GLCM. A fusion of TSBTC with GLCM has provided the best iris presentation attack detection accuracy as 93.78% for the Clarkson 2013 dataset, 95.57% for the Clarkson 2015 dataset, 78.88% for the IIITD Contact dataset, and 99.68% for the IIITD Combined Spoofing datasets. A comparison of the performance of different machine learning classifiers such as naïve Bayes, SVM, random forest, J48, and multilayer perceptron and ensembles of SVM + RF + NB, SVM + RF + RT, RF + SVM + MLP, and J48 + RF + MLP are used for classification accuracy of live and spoof iris detection. J48 + RF + MLP ensembles of classifiers have given a maximum accuracy of 99.68%. Though TSBTC has shown promise in the image classification of colored images for various applications such as land usage identification, gender classification, and so on, it has also shown promising results for the detection of iris presentation attacks. The experimental results showed that the proposed approach efficiently identifies iris spoofing attacks using various sensors.

The feature-level fusion of local GLCM and global TSBTC can distinguish between live and faked artifacts and offer improved outcomes compared to the latest state-of-the-art approaches. The findings show that our proposed approach decreases classification error and improves accuracy compared with the previous approaches used to detect presentation attacks in an iris detection system. This has been tabulated in Table 10. The proposed approach is compared to recent research done in this area, and it has already been concluded that this approach outperforms other methods. Therefore, it works with images of 128 × 128 pixels. Only 10 variations of TSBTC are used during implementation.

## 7. Conclusions

This paper proposed the novel method of ILD to prevent iris spoofing through a textured lens and print attacks. The proposed approach identified both kinds of print attacks (capture/scan) and detected iris spoofing attempted using different sensors. Many approaches have been used in preprocessing as iris segmentation, normalization, and localization, all of which are computationally based on the ILD method. In this research, TSBTC and GLCM features are extracted directly from iris images to overcome this drawback. Feature-level fusions are carried out using global TSBTC and local GLCM features. Various ML algorithms and their ensemble combinations are trained using these fusion features of iris images. The experimental validation of the proposed liveness detection approach is done on four benchmark datasets.

The performance comparison of variants of the proposed approach is made using ISO/IEC biometric performance evolution metrics, including APCER, NPCER, ACER, accuracy precision, recall, and F-measures. For the Clarkson 2013 dataset, fake images are identified with 93.78% accuracy, whereas for Clarkson 2015, the accuracy of the dataset achieved is 95.57% with the RF model. The accuracy obtained for IIITD Contact is 78.88%, and for IIITD Combined Spoofing it is 99.68%. Comparing the performances with Iris Liveness Detection Competition (LivDet-Iris) 2020, the proposed approach got the lowest ACER of 0.48%. The experimental results showed that the proposed approach efficiently identifies iris spoofing attacks using various sensors. In future work, this framework may be extended with the best performance features. Currently, the presented work explored Thepade’s SBTC as a global representation of iris content. However, the local content presentation of Thepade’s SBTC would be an exciting exploration in the future. Moreover, the proposed fusion framework may be applied for the liveness detection of other biometric traits.

## Figures and Tables

**Table 1:** Iris presentation attacks.

Iris Presentation Attacks	Details
Print attacks	The impostor offers a printed image of validated iris to the biometric sensor [4].
Contact lens attacks	The impostor wears contact lenses on which the pattern of the genuine iris is printed [5].
Video attacks	The impostor plays a video of a registered user in front of a biometric system [6].
Cadaver attacks	The impostor uses the eye of a dead person in front of a biometric system [7].
Synthetic attacks	The impostor embeds the iris region into the authentic images to make the synthesized images more realistic [8].

**Table 2:** Summary of literature review.

Paper	Author/Year	Feature Extraction	Attacks Identified	Datasets	Classifiers	Performances
ID						
[17]	Thepade and Chaudhari, 2021	TSBTC and Sauvola thresholding	NA	NR	SVM, Kstar, J48, RF, RT and ensembles	Accuracy, F-measures.
[16]	Dewan and Thepade, 2021	TSBTC	NA	NA	NA	ARA = 63.31%
[12]	Jusman et al., 2020	Hough transform, GLCM	NR	CASIA-Iris	Discriminant analysis classifiers	Accuracy = 100%
[11]	Agarwal et al., 2020	Texture feature, GLCM	Print	ATVs (iris) LivDet2011 (finger) IIITD CLI dataset (iris)	SVM	ACA = 96.3%
[14]	Agarwal et al., 2020	Local binary hexagonal extrema pattern	Contact Print	IIITD CLI ATVS-FIr	SVM	AER = 1.8 %,
[13]	Khuzani et al., 2020	Shape, density, FFT, GLCM, GLDM, and wavelet	NR	CASIA-Iris-Interval	Multilayer neural network	Accuracy = 99.64%
[26]	Kush- waha et al., 2020	GLCM, HOG, LBP	NA	Biometric 220X6 human footprint dataset	KNN, SVM, LDA, ensembles	Accuracy = 97.9%
[22]	Kimura et al., 2020	CNN	Print contact	Clarkson, Warsaw, IIITD-WVU, Notre Dame		APCER = 4.18% BPCER= 0%
[21]	Naqvi et al., 2020	CNN model with a lite-residual encoder–decoder network	NA	NICE-II dataset, SBVPI	CNN	Average segmentation error = 0.0061
[24]	Long and Zeng, 2019	BNCNN	Synthetic, contact	CASIA-Iris-Lamp, CASIA-Iris-Syn, ND contact	BNCNN	Correct recognition rate= 100%
[21]	Asmara et al., 2019	GLCM, Gabor filter		CASIA v1 Iris	Navies Bayes, SVM	Accuracy = 95.24%
[3]	Kaur et al., 2019	Orthogonal rotation-invariant feature set comprising of ZMs and PHTs	Print + scan, print + capture, patterned contact lenses	IIITD-CLI, IIS, Clarkson LivDet-Iris 2015, Warsaw LivDet-Iris 2015	KNN	Accuracy = 98.49% (given different accuracy for different datasets)
[8]	Fathy and Ali, 2018	Wavelet packets (WPs), local binary pattern (LBP), entropy	Print + synthetic	ATVS-Fir CASIA-Iris-Syn	SVM	ACA = 99.92% recall, precision, F1
[18]	Thepade et al., 2018	TSBTC, Niblack	NR	NR	SVM, RF, ensembles, Bayes net	Accuracy = 68.56%
[15]	Thavalen- gal et al., 2016	Pupil localization techniques with distance metrics are used for detection	Print	Real-time datasets	Binary tree classifier	ACER = 0%
[19]	Hu et al., 2016	LBP, histogram, SID	Contact lenses, print	Clarkson, Warsaw, Notre Dame, MobBIOfake	SVM	ER, Clarkson = 7.87%, Warsaw = 6.15% ND = 0.08%, MobBIOfake = 1.50%

**Table 3:** Several images were used for the experiment from each dataset.

Database	Sensor	Image Category	No. of Images Used for the Experiment
Clarkson 2013	Dalsa	Off (live)	350
		Pattern (contact)	440
Clarkson 2015	Dalsa	Live	378
		Pattern	356
		Printed	1416
	LG	Live	258
		Pattern	433
		Printed	844
IIITD Combined Spoofing	Cogent	Normal	2024
		Print-capture	1113
		Print-scan	980
	Vista	Normal	2024
		Print-capture	1092
		Print-scan	1196
IIITD Contact	Cogent	Normal	422
		Transparent	1131
		Textured	1150
	Vista	Normal	1010
		Transparent	1010
		Textured	1010

**Table 4:** Performance evaluation using accuracy for variants of the proposed approach of ILD with N-ary TSBTC and ML classifiers used for the Clarkson 2013 dataset.

Classifiers/Ensembles of Classifiers	Accuracy										
	2-ary	3-ary	4-ary	5-ary	6-ary	7-ary	8-ary	9-ary	10-ary	11-ary	AVG
NB	83.52	83.67	83.52	83.96	84.11	84.11	84.11	84.25	84.4	84.4	83.96
J48	86.15	87.6	86.58	88.04	89.5	86.88	88.92	90.08	91.69	88.77	88.38
SVM	86.58	86.73	86.73	86.73	86.73	86.58	86.44	86.44	86.58	86.44	86.62
RF	89.06	93.29	93.14	93.87	**94.16**	93.29	93.14	93.29	93.87	93	93.01
MLP	86.44	86.58	86.44	86.29	86.58	87.9	87.9	87.9	87.6	88.33	87.07
SVM + RF + NB	86.44	86.88	87.17	87.17	87.17	87.46	86.73	86.88	87.17	86.88	87.01
SVM + RF + RT	88.77	93	92.12	92.41	92.56	92.12	91.54	92.12	92.71	92.56	91.93
RF + SVM + MLP	86.58	86.73	86.88	86.88	86.73	87.17	87.17	87.17	87.31	87.46	86.96
J48 + RF + MLP	87.17	89.5	88.62	89.94	90.08	90.08	90.37	91.39	92.27	91.25	89.94
AVG	86.746	88.22	87.91	88.37	88.62	88.4	88.48	88.84	**89.29**	88.79	——

Bold values indicate the highest obtained recognition rates.

**Table 5:** Performance evaluation using accuracy for variants of the proposed approach of ILD with the N-ary TSBTC and ML classifiers used for the Clarkson 2015 dataset.

Classifiers/Ensembles of Classifiers	Accuracy										
	2-ary	3-ary	4-ary	5-ary	6-ary	7-ary	8-ary	9-ary	10-ary	11-ary	AVG
NB	64.85	64.71	64.44	64.57	64.16	64.16	64.03	63.89	63.89	63.89	64.26
J48	75.61	80.79	82.15	85.96	87.87	89.1	90.05	90.32	91.41	91.28	86.45
SVM	57.08	58.17	59.4	61.17	60.49	60.62	60.89	61.3	61.44	61.98	60.25
Random Forest	83.78	89.64	91.96	94.27	94.68	95.5	94.95	95.5	**95.64**	95.23	93.12
MLP	77.11	78.61	78.47	74.25	80.24	76.02	88.82	89.1	89.23	91.14	82.3
SVM + RF + NB	66.07	68.39	70.7	73.56	73.29	74.65	73.97	75.61	76.15	76.15	72.85
SVM + RF + RT	83.78	88.14	90.73	92.23	92.77	92.5	93.46	93.73	91.96	93.86	91.32
RF + SVM + MLP	78.74	79.15	81.6	76.7	73.43	74.38	70.02	75.34	68.8	71.66	74.98
J48 + RF + MLP	82.28	85.83	87.32	88.96	90.05	92.09	91	92.91	94	94	89.84
AVG	74.367	77.048	78.53	79.07	79.66	79.89	80.8	81.97	81.39	**82.13**	——

Bold values indicate the highest obtained recognition rates.

**Table 6:** Performance evaluation using accuracy for variants of the proposed approach of ILD with the N-ary TSBTC and ML classifiers used for the IIITD Contact dataset.

Classifiers/Ensembles of Classifiers	Accuracy										
	2-ary	3-ary	4-ary	5-ary	6-ary	7-ary	8-ary	9-ary	10-ary	11-ary	AVG
NB	46.41	45.82	44.32	43.88	43.73	44.02	44.47	63.91	63.82	64	50.438
J48	64.47	62.08	64.47	61.19	64.77	62.98	63.88	66.37	65.4	65.58	64.119
SVM	61.04	61.04	61.04	61.04	61.04	61.04	61.04	64.09	64.09	64.09	61.955
Random Forest	63.88	67.61	71.64	70.44	75.52	74.92	75.97	75.41	76.29	**76.73**	72.841
MLP	57.61	62.53	59.7	59.7	61.49	61.79	62.38	65.58	65.23	66.11	62.212
SVM + RF + NB	60.89	60.74	61.49	61.94	62.53	63.88	65.22	65.67	65.58	65.75	63.369
SVM + RF + RT	61.64	66.71	71.04	68.35	71.94	71.04	74.62	73.22	73.57	75.68	70.781
RF + SVM + MLP	61.04	62.08	61.79	61.64	62.08	61.64	61.94	66.28	66.19	66.37	63.087
J48 + RF + MLP	61.49	65.82	68.35	66.86	68.05	68.5	68.95	68.48	68.48	69.71	67.369
AVG	59.83	61.603	62.648	61.671	63.461	63.312	64.274	67.667	67.627	**68.22**	——

Bold values indicate the highest obtained recognition rates.

**Table 7:** Performance evaluation using accuracy for variants of the proposed approach of ILD with N-ary TSBTC and the ML classifiers used for IIITD Combined Spoofing dataset.

Classifiers Ensembles of Classifiers	Accuracy										
	2-ary	3-ary	4-ary	5-ary	6-ary	7-ary	8-ary	9-ary	10-ary	11-ary	AVG
NB	90.09	94.99	95.1	95.2	94.99	94.99	94.88	95.2	95.2	92.49	94.31
J48	98.08	98.29	98.61	98.61	98.08	99.25	99.04	98.93	99.14	98.8	98.68
SVM	96.48	96.59	96.8	97.01	97.01	97.12	97.12	97.44	97.55	97.74	97.08
Random Forest	97.97	98.61	98.82	99.25	99.14	99.46	99.36	99.36	99.25	99.18	99.04
MLP	98.93	99.14	99.25	99.14	99.14	99.14	99.04	99.04	99.04	98.78	99.06
SVM + RF + NB	96.27	96.59	96.91	97.01	97.01	97.23	97.23	97.55	97.65	98.19	97.16
SVM + RF + RT	97.87	98.4	98.5	99.04	99.04	99.25	99.14	99.25	99.04	99.17	98.87
RF + SVM + MLP	98.61	98.72	98.72	98.93	98.93	98.93	98.93	98.93	99.04	98.19	98.79
J48 + RF + MLP	98.4	98.72	98.82	99.04	99.04	**99.57**	99.36	99.25	99.36	99.15	99.07
AVG	96.96	97.78	97.94	98.13	98.04	98.32	98.23	98.32	**98.36**	97.96	——

Bold values indicate the highest obtained recognition rates.

**Table 8:** Performance comparison of GLCM, TSBTC, and fusion of TSBTC and GLCM across all classifiers using an average percentage of accuracy, precision, recall, and F-ratio values.

Classifiers/Ensembles of Classifiers	Accuracy in Percentage (%)											
	Clarkson 2013			Clarkson 2015			IIITD Contact			IIITD Combined Spoofing		
	TSBTC + GLCM	GLCM	TSBTC	TSBTC + GLM	GLCM	TSBTC	TSBTC + GLCM	GLCM	TSBTC	TSBTC + GLCM	GLCM	TSBTC
NB	83.74	81.77	84.40	65.33	67.84	63.89	58.91	59.51	63.91	97.55	92.86	93.85
J48	91.54	83.23	90.23	91.34	73.97	91.35	69.31	72.94	65.49	99.04	96.43	98.97
SVM	86.73	82.21	86.51	61.99	59.94	61.71	64.09	73.20	64.09	98.99	95.51	97.65
Random Forest	**93.78**	82.79	93.44	**95.57**	82.01	95.44	**78.88**	75.36	76.51	99.57	96.73	99.22
MLP	88.26	83.81	87.97	90.30	77.11	90.19	64.13	73.01	65.67	99.57	98.06	98.91
SVM + RF + NB	86.88	83.38	87.03	79.43	64.98	76.15	69.18	73.64	65.67	99.20	96.63	97.92
SVM + RF + RT	91.84	82.50	92.64	94.34	81.33	92.91	76.42	74.79	74.63	99.57	97.04	99.11
RF + SVM + MLP	87.61	83.81	87.39	82.49	75.47	70.23	67.51	73.32	66.28	99.62	97.55	98.62
J48 + RF + MLP	92.63	83.81	91.76	94.00	79.70	94.00	76.07	74.72	69.10	**99.68**	97.34	99.26
AVG	89.22	83.03	89.04	83.86	73.59	81.76	69.39	72.28	67.93	99.20	96.46	98.16

Bold values indicate the highest obtained recognition rates.

**Table 9:** Performance evaluation using accuracy for the proposed approach of ILD for various datasets used during implementation.

Datasets	Classifiers	Accuracy in %	Precision in %	Recall in %	F-Measure in %	APECR in %	NPCER in %	ACER in %
Clarkson 2013	Random Forest	93.78	95.50	86.20	90.60	7.90	4.12	6.01
Clarkson 2015	Random Forest	95.57	96.50	95.50	96.00	4.72	3.47	4.09
IIITD_Contact	Random Forest	78.88	79.30	79.40	78.60	21.56	20.28	20.92
IIITD_Spoofing	J48 + RF + MLP	**99.68**	**99.80**	**99.80**	**99.80**	**0.12**	**0.84**	**0.48**

Bold values indicate the highest obtained recognition rates.

**Table 10:** The comparative analysis/study of the proposed approach and prevailing methods.

Author/Year	Feature Extraction	Dataset	Performance Measure	Classifiers	Results (%)
P. Das et al., 2021 [39]	MSU PAD1 MSU PAD2 Notre Dame PAD	Clarkson University (CU), University of Notre Dame (ND), and Warsaw University of Technology (WUT)	APCER, BPCER, ACER	SVM, RF, MLP and CNN.	ACER = 2.61 ACER = 2.18 ACER = 28.96
Arora et al., 2021 [40]	CNN	IIITD	Accuracy FAR	VGGNet	Acc = 97.98
				LeNet	Acc = 89.38
				ConvNet	Acc = 98.99
Omran and Alshemmary 2020 [41]	CNN, IRISNet	IIITD	Sensitivity, accuracy, specificity, precision recall, G mean, and F-measure	(SVM, KNN, NB, DT	Acc = 96.43
Zhao et al., 2019 [42]	Mask R-CNN	IIITD	Accuracy	R-CNN, CNN	Acc = 98.9
Wang and Kumar 2019 [43]	CNN-SDH, CNN-Joint Bayesian	PolyU bi-spectra	Accuracy	CNN, SDH	Acc = 90.71
Cheng et al., 2019 [44]	CNN	CASIA-Iris-L	Accuracy	Hadamard + CNN	Acc = 97.41
Chatterjee et al., 2019 [45]	DWT, ResNet	ATVS	Accuracy	ResNet	Acc = 92.57
Proposed Approach	TSBTC, GLCM, Fusion of TSBTC and GLCM	Clarkson 2013	Accuracy, precision, recall, and F-measure	Random Forest	Acc = 93.78
		Clarkson 2015		Random Forest	Acc= 95.57
		IIITD Contact		Random Forest	Acc = 78.88
		IIITD Combined Spoofing		J48 + RF + MLP	Acc = 99.68 ACER = 0.48

## Data Availability

The data supporting this study’s findings are available on request from the corresponding author. The data are not publicly available due to the privacy concern of research participants.
